# Comments on “The effect of age on third molar extraction difficulty: a retrospective cross-sectional cohort study” – Danger in describing ‘simplicity’ in third molar surgery

**DOI:** 10.2340/aos.v85.45933

**Published:** 2026-04-29

**Authors:** Aydin Gülses

**Affiliations:** Department of Oral and Maxillofacial Surgery, University Hospital Schleswig-Holstein, Kiel, Germany

I read with great interest the study by Noro et al., which analyzed 12,649 third molar extractions in Helsinki. The authors report that while surgical extractions and simple maxillary extractions become increasingly demanding with age, mandibular extractions appeared to involve lower technical complexity in older patients [1].

I believe that this observed lower apparent difficulty in mandibular extractions warrants further exploration of underlying anatomical and physiological factors, particularly bone quality. In Northern Europe, including Finland, older adults – particularly postmenopausal women – often exhibit reduced bone mineral density (BMD), with densitometric osteoporosis observed in ~21.5% of women and ~6.4% of men aged ≥ 50 years [2]. Emerging evidence suggests that preoperative Cone Beam Computed Tomography (CBCT)‑derived mandibular BMD values correlate with surgical difficulty in impacted mandibular third molar extraction, with higher BMD independently predicting increased operative challenge [3].

Although noteworthy, a mandibular extraction demonstrating technical simplicity should not be equated with clinical safety. Age-related declines in endosteal bone healing, systemic comorbidities, and concomitant medications can impair wound healing and increase the risk of adverse outcomes, including medication-related osteonecrosis of the jaw (MRONJ). Moreover, matched-pair analyses comparing elderly patients (≥ 65 years) with younger adults demonstrate a significantly greater burden of peri- and postoperative morbidity in the older cohort, including higher rates of ankylosis, nerve proximity, and complications necessitating longer operative times and hospital stays, suggesting that delaying extraction into late adulthood may shift the morbidity burden to later life [4, 5].

Therefore, planning third molar removal in older adults should integrate anatomical evaluation with a comprehensive assessment of physiologic, pharmacologic, and systemic risk factors, rather than relying solely on perceived technical simplicity or surgical complexity. Recognizing these limitations is essential to ensure safe and predictable outcomes and to avoid overinterpreting mandibular extraction performance in aging populations.
